# Relationship between VacA Toxin and Host Cell Autophagy in *Helicobacter pylori* Infection of the Human Stomach: A Few Answers, Many Questions

**DOI:** 10.3390/toxins8070203

**Published:** 2016-07-01

**Authors:** Vittorio Ricci

**Affiliations:** Department of Molecular Medicine, Human Physiology Unit, University of Pavia Medical School, Via Forlanini 6, 27100 Pavia, Italy; vricci@unipv.it; Tel.: +39-0382-987254

**Keywords:** VacA vacuolating toxin, autophagy, *Helicobacter pylori*, gastric cancer

## Abstract

*Helicobacter pylori* is a Gram-negative bacterium that colonizes the stomach of about half the global population and represents the greatest risk factor for gastric malignancy. The relevance of *H. pylori* for gastric cancer development is equivalent to that of tobacco smoking for lung cancer. VacA toxin seems to play a pivotal role in the overall strategy of *H. pylori* towards achieving persistent gastric colonization. This strategy appears to involve the modulation of host cell autophagy. After an overview of autophagy and its role in infection and carcinogenesis, I critically review current knowledge about the action of VacA on host cell autophagy during *H. pylori* infection of the human stomach. Although VacA is a key player in modulation of *H. pylori*-induced autophagy, a few discrepancies in the data are also evident and many questions remain to be answered. We are thus still far from a definitive understanding of the molecular mechanisms through which VacA affects autophagy and the consequences of this toxin action on the overall pathogenic activity of *H. pylori*.

## 1. Introduction

*Helicobacter pylori* is a Gram-negative bacterium that colonizes the stomach of about half the global population, making it one of the most common bacterial infections worldwide [1,2,3,4]. If untreated, the infection becomes chronic and persists throughout life despite a vigorous humoral and cellular immune response [2,4]. *H. pylori* infection always causes active chronic gastritis that can remain clinically silent for several decades after initial infection, due to the dynamic equilibrium between the bacterium and its human host. *H. pylori* infection may also evolve into more severe diseases, such as atrophic gastritis, peptic ulcer, lymphoma of the mucosa-associated lymphoid tissue, or gastric adenocarcinoma [1,3,5,6,7]. In 1994, *H. pylori* was classified by the World Health Organization as a class I carcinogen [8].

According to the most recent global estimate (GLOBOCAN 2012), despite its declining incidence rate, gastric cancer remains the fifth most common malignancy worldwide and the third leading cause of cancer-related mortality, with >700,000 deaths annually [9]. *H. pylori* infection is the strongest known risk factor for gastric malignancy. The attributable risk for developing cancer of the distal, non-cardia stomach conferred by *H. pylori* is approximately 89% [10]. Thus, the relevance of *H. pylori* for gastric cancer development is similar to that of tobacco smoking for lung cancer [3].

Development of gastric adenocarcinoma occurs, however, only in a small percentage of *H. pylori*-infected individuals [2,11]. *H. pylori*-related gastric carcinogenesis appears to result from a well-choreographed interaction between the pathogen and its host, which depends on strain-specific bacterial factors, host genotypic traits, and permissive environmental factors [2,3].

Among *H. pylori* virulence factors, a key role is played by vacuolating toxin VacA and the effector protein CagA, encoded by the 40-kbp chromosomal *cag* pathogenicity island together with a type IV secretion system that injects CagA into the host cell [2,3,5,7,11,12]. VacA seems to play a pivotal role in the overall strategy of *H. pylori* towards establishing persistent colonization of the hostile gastric environment that represents its ecological niche [12,13]. Mounting evidence suggests that such a strategy may involve the modulation of host cell autophagy (reviewed in [14,15]).

In this review, after an overview of autophagy and its role in infection and carcinogenesis, I critically assess current knowledge about the effects of VacA on host cell autophagy during *H. pylori* infection of the human stomach.

## 2. Autophagy

The Greek-derived term “autophagy”, coined by C. de Duve (the discoverer of the lysosome) in 1963, literally means “self-eating” and describes an evolutionarily highly-conserved process through which cellular components are degraded by lysosomes (reviewed in [16,17]). However, three different pathways can deliver intracellular cargo to lysosomes for degradation: macroautophagy, microautophagy, and chaperone-mediated autophagy [18,19]. In macroautophagy, intracellular cargoes are first sequestered inside double-membrane vesicles, referred to as autophagosomes, which then undergo fusion with lysosomes to form autolysosomes (also called autophagolysosomes). In microautophagy, the lysosomes themself, through invagination and pinching off of their limiting membranes, directly take up and degrade cytoplasmic components. In chaperone-mediated autophagy, specific cytoplasmic proteins (i.e., those containing the KFERQ pentapeptide sequence) are directly translocated across the lysosomal membrane in a complex with chaperone proteins (e.g., heat shock cognate protein-70). In the present review, I focus on macroautophagy (hereafter simply referred to as autophagy), and its key roles in human health and disease, including cancer and *H. pylori* infection [14,15,16,17,20].

Autophagy was classically conceived as a physiological process that maintains cellular homeostasis, acting as a quality and quantity control process by selectively removing protein aggregates and damaged or old or surplus organelles (such as mitochondria, ribosomes and endoplasmic reticulum), as well as invading microbial pathogens [17,21]. Stress and starvation strongly induce autophagy. Bulk degradation of cytoplasmic components through autophagy provides the cell with metabolic substrates (released from autolysosomes and thus recycled in the cytosol), supporting the heightened bioenergetic and biosynthetic needs of the cell [16,17,20,21]. This is a cytoprotective response resulting in cell adaptation and survival. However, dysregulated or excessive autophagy may result in cell death [17,20].

### 2.1. Molecular Machinery of Autophagy

**Figure 1:**
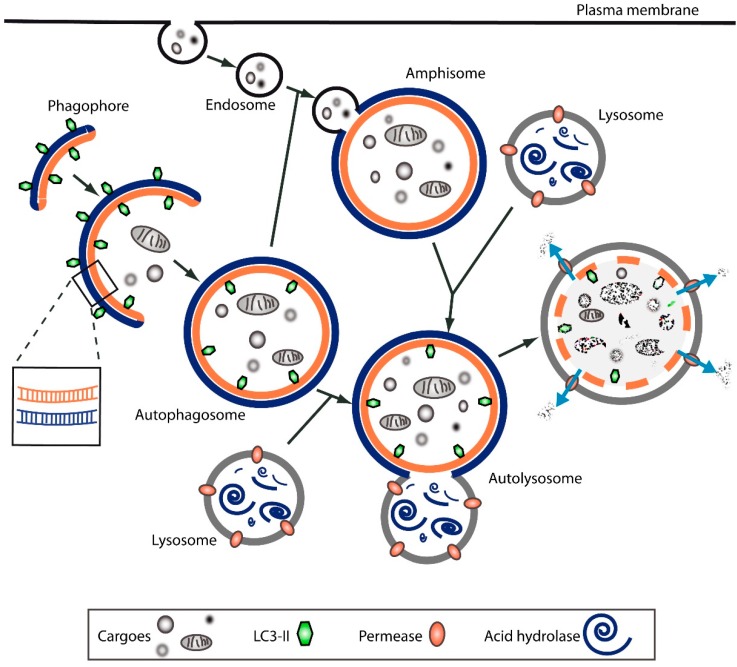
Scheme of autophagy as a multi-step process. See text for details.

Autophagy can be envisaged as a multi-step process (Figure 1). It is initiated by the formation of an isolation membrane, also known as a phagophore; a double-layered, crescent-shaped membrane whose exact origin is still debated [17,18,22,23]. However, several lines of evidence suggest that the endoplasmic reticulum may serve as the main source of the autophagosome or as a platform for autophagosome formation [22,24]. The phagophore elongates and expands to engulf, selectively or not, intracellular cargoes, which are finally enclosed in the double-membrane autophagosome. This matures through docking and fusion with an endosome (to form the so-called amphisome) and/or a lysosome (to form the autolysosome), where the autophagosome inner membrane and cargo are degraded through acid hydrolases. The resultant macromolecules are recycled back into the cytosol through permeases. Together, all these steps form the so-called autophagic flux.

More than 35 autophagy-related genes (*ATG*) encode for the overall autophagy machinery [24]. These genes were originally identified in yeast but are highly conserved in humans. A subset of approximately 18 Atg proteins is essential for formation of canonical autophagosomes and is thus referred to as the core molecular machinery of autophagy [17,19,24]. Upon induction of autophagy (in which inhibition of the mammalian target of rapamycin, mTOR, plays a key role), these proteins associate in a well-defined hierarchical order. They have been classified in four functional groups (each including several proteins): (1) Atg1/ULK complex (formed by ULK1/2, Atg13, FIP200 and Atg101 in mammals); (2) phosphatidylinositol 3-kinase (PI3K) complex; (3) Atg9 trafficking system; and (4) two ubiquitin-like proteins (Atg12 and microtubule-associated protein 1 light chain 3, commonly known as LC3, which is a mammalian homologue of Atg8) and their respective, partially overlapping, conjugation systems.

The lipidated form of LC3 (named LC3-II) is extensively used to monitor autophagy (either by fluorescence microscopy as LC3 puncta or by Western blotting because of its increased electrophoretic mobility in SDS-PAGE compared to the nonlipidated form, LC3-I). LC3-II is localized in autophagic structures throughout the process from phagophore elongation to lysosomal degradation [14,15,25]. After translation, the unprocessed form of LC3 (proLC3) is proteolytically cleaved by Atg4 to the cytosolic LC3-I form, which exhibits a glycine at its C terminus. Upon induction of autophagy, through the highly coordinated action of different factors (i.e., Atg7, Atg3, and Atg12-Atg5-Atg16L1 multimers) such a glycine is conjugated to phosphatidylethanolamine to generate LC3-II. The highly lipophilic phosphatidylethanolamine moiety promotes LC3-II attachment to both faces of the phagophore membrane, where it plays a role in membrane fusion and selecting cargo for degradation [18,19,25]. In selective autophagy, LC3-II acts as a receptor at the phagophore, interacting with adaptor molecules (such as p62/sequestosome 1). These molecules are bound to tagged (e.g., ubiquitinated) targets, such as protein aggregates or damaged mitochondria, to promote their selective uptake, and thus degradation, by the autophagic machinery [18,19]. LC3-II bound to the autophagosome outer membrane is ultimately delipidated and recycled, whereas LC3-II bound to the autophagosome inner membrane is degraded by lysosomal hydrolases [15,17].

However, it is widely accepted that measuring autophagy in a meaningful way requires an analysis of the rate of autophagic flux (i.e., the rate at which material is cleared from the cell by autophagy). Thus, steady-state LC3-II levels alone are not sufficient and may be misleading for evaluating autophagy [14,16,25,26]. In addition, LC3 can aggregate within cells or be incorporated in inclusion bodies in an autophagy-independent manner or be recruited to macroendocytic vacuoles (including macropinosomes, phagosomes, and entotic vacuoles) in a pathway distinct from canonical autophagy [14,26,27,28]. In particular, LC3-II can be recruited to single-membrane phagosomes in a process known as LC3-associated phagocytosis, which has been suggested to result in more efficient phagosome maturation than conventional phagocytosis does [29,30].

### 2.2. Autophagy and Bacterial Infection

Beyond its original meaning of self-eating, autophagy is able to target invading pathogens, especially bacteria. This type of selective autophagy is termed xenophagy [24,29,31,32]. A variety of host mechanisms recognize and deliver intracellular bacteria to autophagosomes for degradation. Autophagy can be envisaged as an evolutionarily conserved innate immune response of the host to infection, and defects in the process result in increased susceptibility to infectious diseases [29,31,32,33]. However, as well underlined by Choy and Roy [32], in a constantly evolving “arms race”, bacteria have evolved a variety of mechanisms to counteract this immune response so as to remain one step ahead of the host cell. These bacterial strategies include: (1) evasion of autophagy; (2) inhibition of autophagy; and (3) subversion of autophagy (i.e., hijacking it so as to favor bacterial survival and replication) (reviewed in [29,32,33]).

Although *H. pylori* is commonly considered to be an extracellular, noninvasive bacterium, increasing evidences suggest that it can invade, survive and multiply in epithelial and immune cells of the human stomach (reviewed in [14,15]). The ability of *H. pylori* to create an intracellular niche may contribute to infection persistence, evasion of the host immune response, and resistance to eradication by membrane-impermeable antibiotics [14]. Autophagy appears to be initially stimulated as a host attempt to eliminate the invading pathogen. In epithelial cells and macrophages, intracellular survival and replication of *H. pylori* are favored by autophagy inhibition through 3-methyladenine and, on the contrary, counteracted by stimulation of autophagy through rapamycin [34,35]. Moreover, people carrying the *ATG16L1T300A* Crohn’s disease risk allele (which is suggested to result in impaired xenophagy) are more susceptible to *H. pylori* infection (see below) [15,36]. Nevertheless, in its battle for survival, *H. pylori* is able to inhibit autophagy directly and subvert it so as to favor establishment of its intracellular replication niche [14,15]. *H. pylori* (especially its most virulent strains) has been shown to downregulate expression of key autophagic proteins [35,37]. However, as mentioned above, in the interplay between *H. pylori* and host cell autophagy, VacA toxin has a key role.

### 2.3. Autophagy and Cancer

It is well known that autophagy can exert both tumor-suppressive and tumor-promoting activities (reviewed in [20,38]) and this may create some confusion. However, as emphasized by Galluzzi et al. [38], one must keep in mind the aforementioned key role of autophagy in cell biology: the maintenance of cellular homeostasis. From a cell-centered point of view, this activity has beneficial effects; however, these effects may be detrimental to the host in a context-dependent fashion. In healthy nontransformed cells, the homeostatic activity of autophagy counteracts malignant transformation and is required for efficient anticancer immunosurveillance [38]. Autophagy is inhibited by oncoproteins and stimulated by oncosuppressor proteins [38]. In tumor cells, in contrast, autophagy promotes survival in response to several stressors (such as starvation, growth factor deprivation, hypoxia, and anticancer drugs) that characterize the tumor microenvironment [20,38]. This supports tumor progression, invasiveness and resistance to anticancer therapy.

In healthy cells, autophagy exerts its oncosuppressive action through elimination of oncogenic proteins (such as p62/sequestosome 1), toxic unfolded protein aggregates, and damaged organelles [20,38]. In the latter case, autophagic removal of dysfunctional mitochondria (a process known as mitophagy) prevents oxidative stress by overproduction of reactive oxygen species (ROS), which are highly genotoxic, and thus promotes genomic stability. This action also contributes to the anti-inflammatory effect of autophagy [29,38]. Genomic stability may also be achieved through removal of micronuclei and damaged nuclear parts [39]. Moreover, the oncosuppressive activity of autophagy is contributed by its key role in the defense against invading pathogens (i.e., xenophagy) [29,38]. In other words, efficient autophagy may prevent/limit chronic tissue damage, inflammation and genome instability, which together create a tumor-initiating and tumor-promoting environment [20].

## 3. VacA as an Autophagy Inducer

Soon after VacA was discovered by Leunk et al. [40], through evidence of its ability to cause formation of large cytoplasmic vacuoles in cultured mammalian cells, pioneering studies suggested an autophagic origin of VacA-induced vacuoles [41,42]. This hypothesis was mainly based on the fact that: (1) the vacuoles were acidic (indicated by accumulation of neutral red and acridine orange) and contained acid phosphatase; (2) in the early stages of their formation, they were not labeled by fluid-phase endocytic markers such as Lucifer yellow; and (3) in the mature stages, they showed a luminal content represented by degenerate cytoplasmic components, suggestive of autolysosomes. However, there was no ultrastructural evidence of double-membrane autophagosomes. Now, it is widely accepted that: (1) VacA-induced vacuoles derive from an acidic late endosomal compartment where the toxin localizes after internalization; (2) such a compartment is distinct from autophagosomes; and (3) VacA induces formation of a hybrid compartment with markers of late endosomes and lysosomes, but with a reduced proteolytic activity [13,43,44,45,46,47,48,49,50]. Vacuoles virtually identical to those observed in VacA-treated cultured cells are also found in surface gastric epithelial cells in gastric biopsies from *H. pylori*-infected patients [50,51,52]. Nevertheless, the role of VacA-induced cell vacuolation in the overall pathogenic action of *H. pylori* remains unclear.

Jones and coworkers showed that VacA promoted *H. pylori* survival in macrophage and gastric epithelial cell lines [53,54], and subsequently investigated whether VacA affected autophagy in AGS human gastric epithelial cell line infected with *H. pylori* [49]. Autophagy was monitored by transmission electron microscopy (TEM) and by detection of the lipidated form of LC3 (LC3-II) by Western blotting and as a punctate pattern in fluorescence microscopy. Terebiznik et al. [49] found that *H. pylori* triggered autophagy with the appearance of autophagosomes, different in size and morphology from typical VacA-induced vacuoles; some of which contained bacterial bodies. To confirm that this phenomenon was dependent upon canonical autophagy, they infected Atg5-knockout mouse embryo fibroblasts (MEFs) with *H. pylori* and observed a decrease in LC3 puncta formation compared to that in wild-type MEFs. Bacterial components/virulence factors such as urease and CagA, as well as its type IV secretion machinery, appeared to be dispensable in *H. pylori*-induced autophagy. In contrast, VacA was the bacterial virulence factor that was necessary and sufficient to induce autophagy. This was demonstrated by infecting AGS cells with *vacA*-defective isogenic mutant *H. pylori* or by incubating the cells with bacterial broth culture filtrates (BCFs) containing or not VacA or with purified VacA. Similar to VacA-induced vacuolation, VacA-induced LC3-II formation and LC3 punctate pattern were strictly dependent on the channel-forming activity of the toxin. This was demonstrated using BCFs from isogenic mutant strains not producing an actively vacuolating toxin (i.e., P9A or G14A *vacA* mutants from *H. pylori* strain 60190) or the inactive s2m2 allelic form of VacA (from *H. pylori* strain Tx30A). VacA also partially colocalized with LC3 puncta, suggesting that the toxin is associated with autophagosomes. However, the pathway by which such VacA molecules enter the autophagic compartments remains to be established. Terebiznik et al. [49] also found that, while Atg12-knockdown AGS cells exhibited less VacA-induced LC3 puncta and LC3-II generation than control cells, such cells also displayed increased VacA-dependent vacuolation. These results suggest that, even though VacA-induced vacuole genesis is independent from autophagy, autophagy may play a role in counteracting vacuole development. This finding well fits with the observation by Tang et al. [35] that *H. pylori* infection of AGS cells caused increased expression of miRNA *MIR30B*, which downregulated autophagy by inhibiting Atg12 and Beclin-1 expression. This resulted in an increase in intracellular survival of the bacterium and VacA-dependent formation of large vacuoles. Terebiznik et al. [49] speculated that the inhibition of autophagy may increase the stability of intracellular VacA, which in turn results in increased cell vacuolation. Indeed, after a 6-h pulse of VacA followed by a 24-h chase, control AGS cells showed time-dependent degradation of intracellular VacA associated with disappearance of cell vacuoles. However, virtually no VacA degradation was evident in Atg12-knockdown AGS cells in which vacuolation was still present after a 24-h chase. According to Terebiznik et al. [49], induction of autophagy by VacA may represent a host mechanism to limit the extent of toxin-induced cell damage by increasing VacA intracellular degradation, thereby conferring protection to host cells against *H. pylori* infection. Nevertheless, previous studies have suggested that, once internalized, VacA persists inside cultured epithelial cells for many hours with little noticeable degradation and without loss of its vacuolating power [55,56,57].

Based on the observations of Terebiznik et al. [49], the molecular pathways through which VacA may cause autophagy have been investigated by Yahiro et al. [58] in the AZ-521 cell line (until recently regarded as a gastric epithelial cell line, but now known to be a misidentified HuTu-80 cell line derived from human duodenal carcinoma [59]). Autophagy was monitored by detection of the lipidated form of LC3 (LC3-II) by Western blotting and as a punctate pattern in fluorescence microscopy. Yahiro et al. [58] found that both VacA-dependent autophagy and apoptosis were strictly dependent on VacA internalization after binding to a newly identified receptor: low-density lipoprotein receptor-related protein-1 (LRP1), which in AZ-521 cells apparently plays a key role in toxin binding and internalization leading to vacuole development. According to Tsugawa et al. [60], LRP1 seems to bind to only the m1 VacA genotype (reviewed in [12,13]), that is, one associated with a higher degree of epithelial damage, gastritis severity, gastric atrophy, precancerous intestinal metaplasia and increased risk for development of gastric cancer in *H. pylori*-infected patients [11,13]. Confocal microscopy analysis showed that internalized VacA partially colocalized with LC3 puncta and LRP1. LRP1 silencing by siRNA abolished such colocalization as well as VacA-dependent conversion of LC3-I to LC3-II, as shown by Western blotting. In contrast, silencing of other VacA receptors, such as RPTPα and β, which are present on the plasma membrane of AZ-521 cells and able to trigger VacA internalization leading to vacuolation, had no effect on VacA-dependent conversion of LC3-I to LC3-II. Confocal microscopy revealed that VacA-induced vacuoles were at least of two types: LC3-II positive (and thus defined as autophagic) and LC3-II negative. The latter type showed VacA colocalization with either RPTPα or β. These observations fit with previous findings that VacA-induced autophagosomes and large vacuoles are distinct and different intracellular structures [49]. Internalized VacA is known to reach and damage mitochondria (causing mitochondrial membrane depolarization and cytochrome *c* release) [12,61], thus potentially triggering mitophagy; the selective form of autophagy targeting damaged mitochondria to limit cell damage and prevent cell death [20,38,62]. However, Yahiro et al. [58] did not observe mitochondria inside VacA-induced LC3-II-positive vacuoles. In addition to LC3-I to LC3-II conversion, silencing of either LRP1 or Atg5 also inhibited VacA-induced cleavage of caspase 7 and poly(ADP-ribose) polymerase (PARP); both markers of apoptosis. In contrast, chemical inhibition of apoptosis did not affect VacA-induced autophagy, which thus appears to precede apoptosis in AZ-521 cells. Nevertheless, Radin et al. [63] showed that, in AZ-521 cells, VacA did not cause PARP cleavage and that VacA-induced cell death was accounted for by programmed necrosis (also known as necroptosis). In agreement with previous observations [49], using chloride channel blockers (NPPB and DIDS) that impair VacA channel activity and cell vacuolation, Yahiro et al. [58] found that VacA channel activity was required for LRP1-dependent LC3-II generation in response to VacA in AZ-521 cells. However, this was not apparently the case in AGS cells. In AZ-521 cells, VacA-induced autophagy did not involve the canonical pathway, showing that neither Beclin-1 silencing nor 3-methyladenine (which inhibits class 3 PI3K) treatment affected LC3-II generation triggered by the toxin.

People carrying a Crohn’s-disease-associated single nucleotide polymorphism in the autophagy gene *ATG16L1* (i.e., rs2241880, which leads to threonine-to-alanine substitution at position 300) had increased susceptibility to *H. pylori* infection associated with impaired autophagic response to VacA [36]. In two separate cohorts, Raju et al. [36] demonstrated a positive correlation between the presence of the *ATG16L1T300A* risk allele and not just *H. pylori* infection itself but infection with the more toxigenic *H. pylori* strains. When peripheral blood monocytes isolated from healthy volunteers carrying the 300A risk allele were exposed to VacA, reduced LC3-II generation was observed in comparison to cells isolated from individuals carrying the 300T non-risk allele [36].

### Autophagy in the Functional Relationship between VacA and CagA

Mounting evidence suggests the existence of a functional relationship between VacA toxin and the bacterial oncoprotein CagA during *H. pylori* infection (reviewed in [12,13]). Although CagA activates the transcription factor NFAT in cultured gastric epithelial cells, VacA inhibits it. While CagA decreases VacA-induced vacuolation, VacA reduces CagA-induced unique elongation of cultured cells (i.e., the so-called hummingbird phenotype). VacA downregulates the effects of CagA on epithelial cells by interfering with epidermal growth factor receptor activation and endocytosis. CagA counteracts VacA apoptotic activity, impairing VacA internalization and intracellular trafficking, as well as stimulating antiapoptotic gene expression. In polarized epithelial cell monolayers, VacA and CagA may act in concert to provide specific nutrients such as iron to *H. pylori* attached to the apical cell pole. Such cooperative action between CagA and VacA could allow *H. pylori* to use the apical surface of the human gastric epithelium as a replicative niche.

The functional relationship between VacA and CagA is an intriguing strategy to achieve the best bacterial fitness with the hostile gastric environment, avoiding excessive cell damage and optimizing bacterial growth. This might explain the almost constant association of active VacA with CagA in highly pathogenic *H. pylori* (i.e., the so-called type I strains), even though the respective genes are distant on the chromosome and their expression levels are not mutually related [1,12,64]. This relationship could be an additional example of the emerging concept that, in bacterial pathogenesis, virulence factors can regulate each other by functioning as a “team” together or antagonizing each other by “friendly fire” to improve colonization and propagation within the host [13,65].

In *H. pylori*-infected AGS cells undergoing bacterial eradication 5 h post-infection, intracellular CagA underwent degradation (detected most clearly at 15 and 24 h after eradication) by autophagy specifically triggered by m1-type VacA through interaction with LRP1 receptor [60]. In contrast to previous findings in AZ-521 cells [58], LC3-II generation and CagA autophagic degradation induced by VacA were inhibited by 3-methyladenine treatment. Autophagy-mediated degradation of CagA was confirmed in MKN-28-derived WT-A10 cells (in which CagA expression was induced through the pTet-off-*cagA* expression vector) after 24 h treatment with autophagy-inducer rapamycin, VacA-containing *H. pylori* BCFs, or purified VacA. TEM analysis of rapamycin-stimulated WT-A10 cells revealed the presence of immunogold-labeled CagA in autophagic vesicles. In CagA-expressing WT-A10 cells, both m1- and m2-type VacA caused cell vacuolation in a dose-dependent manner, although the effect was stronger with m1 VacA. The fact that m2 VacA was not associated with CagA degradation nor LC3-II generation , confirmed that VacA-induced autophagy is independent of the vacuolating activity of the toxin.

Tsugawa et al. [60] provided many important data about the molecular mechanisms and signaling cascade through which VacA may induce autophagy-mediated degradation of CagA. According to their data, the following scenario can be depicted: (1) through interaction with LRP1 receptor, by a yet unidentified mechanism, VacA decreases intracellular glutathione (GSH) levels, leading to increased ROS production independent of NADPH oxidase or mitochondrial activity; (2) VacA-induced ROS activate Akt through phosphorylation of Ser473; (3) activated Akt stimulates Ser166 phosphorylation of the MDM2 (murine double minute 2 homolog) protein, which increases the ubiquitination and proteasomal degradation of p53; and (4) p53 degradation exerts rapamycin-like induction of autophagy, finally resulting in CagA degradation. However, it remains to be established how CagA is (selectively?) targeted by the autophagic machinery.

Tsugawa et al. [60] observed that CagA can accumulate, thus exerting a sustained carcinogenic activity, in gastric epithelial cells expressing the cell surface marker CD44v9. This is typically associated with cancer stem-like cells and can be induced in normal gastric epithelium by chronic inflammation triggered by *H. pylori* infection [66,67]. In CD44v9-positive cells, VacA-induced autophagy of CagA is suppressed; apparently because these cells are highly resistant to ROS generation due to their increased intracellular GSH synthesis.

## 4. VacA as an Autophagy Inhibitor

It has been shown that limited exposure (i.e., 6 h) of AGS cells to VacA results in increased autophagy with degradation of the toxin [49]. Therefore, Raju et al. [36] decided to investigate the effects of more prolonged exposure (i.e., up to 24 h) of AGS cells to VacA to mimic conditions in chronic infection. The autophagic flux was studied using cells transfected with a tandem construct in which LC3 was tagged with green fluorescent protein (GFP) and monomeric red fluorescent protein (mRFP). This allowed the investigators to follow autophagosome maturation because, after autolysosome formation, the mRFP signal persists longer than the GFP one because the former is resistant to autolysosomal proteases. Surprisingly, Raju et al. [36] observed that both the signals persisted, as in the case of cells incubated with vinblastine, which is known to prevent fusion of autophagosomes with lysosomes. This suggests that VacA inhibits autophagy by disrupting the autophagic flux. This finding was corroborated by the observation that autophagy-dependent degradation of long-half-life proteins was significantly lower in VacA-treated cells compared with rapamycin-treated cells. VacA also counteracted rapamycin-induced autophagic cell death, in a manner similar to autophagy disruption achieved through Atg12 silencing. Raju et al. [36] found that VacA caused intracellular accumulation of defective autolysosomes that lacked the key lysosomal hydrolase cathepsin D, whose activity is required to complete autophagic degradation. However, the autolysosomes did have an acidic lumen and the late endosomal/lysosomal marker Lamp1. These are also well-known characteristics of the late endosomal compartment where the toxin localizes after internalization, from which typical VacA-induced large vacuoles originate, and which may serve as an intracellular replicative niche for *H. pylori* [13,45,46,48,49].

As shown by Raju et al. [36], such disrupted autophagy also resulted in accumulation of p62/sequestosome 1 and elevated ROS levels, which both potentially play a role in *H. pylori*-induced carcinogenesis [15,20,38]. An increased level of p62/sequestosome 1 was also found in gastric biopsy samples from patients infected by *H. pylori* strains that produced active VacA in comparison to nontoxigenic strains [36].

Jones and coworkers [15,36,68] proposed a working model in which the action of VacA on the host autophagic machinery differs according to “acute” or “chronic” exposure to the toxin. In acute *H. pylori* infection or with brief (up to 6 h) direct toxin exposure, VacA stimulates autophagy as a cytoprotective reaction to mitigate toxin-induced damage through VacA degradation and eliminate invading bacteria. In contrast, in chronic *H. pylori* infection or with prolonged direct toxin exposure (both of which may result from a reduced host autophagic response like that associated with the *ATG16L1T300A* risk allele), VacA disrupts autophagy. This may facilitate intracellular bacterial survival and persistent infection but may also create a microenvironment promoting inflammation and carcinogenesis. However, the fascinating idea of different effects of VacA on autophagy depending on duration/level of cell exposure to the toxin deserves further investigations, especially to define better its timing and related molecular mechanisms. According to the data of Raju et al. [36] and Satin et al. [46], the timing of VacA-induced loss of cathepsin D activity, which should play a key role in autophagy disruption during chronic bacterial infection/VacA intoxication, seems to be an acute effect. This does not fit with the timing of autophagy-dependent degradation of VacA shown by Terebiznik et al. [49] and CagA in VacA-intoxicated cells observed by Tsugawa et al. [60].

Moreover, a clear understanding of VacA role in autophagy is made even more difficult by the recent findings of Florey et al. [28], which also challenge previous observations about the role of the toxin in causing autophagy. They found that the lysosomotropic weak base chloroquine, which disrupts the autophagic flux and is commonly used to inhibit autophagy in cultured cells, was by itself able to trigger LC3-II generation in endolysosomal compartments. This autophagy-independent process was apparently driven by the osmotic imbalance caused within endolysosomal compartments. This imbalance could combine with V-ATPase activity to lipidate LC3 onto vacuolar membranes through recruitment of the autophagy lipidation machinery (e.g., Atg5), but independently of the autophagy preinitiation complex (e.g., Atg13) and class 3 PI3K. Chloroquine is known to induce cytoplasmic vacuoles that are almost identical to those induced by VacA [69]. In this respect, VacA-induced vacuole development strictly depends on weak bases, such as ammonia produced by *H. pylori* urease, a key enzyme that enables bacterial colonization and survival in the human stomach. VacA by itself is not vacuolating but greatly increases the vacuolating activity of weak bases [47,70]. Since the pioneering studies of Okhuma and Poole in the early 1980s [71,72], weak bases have been known to cause vacuolation in eukaryotic cells. They cross cell membranes in an uncharged state and are trapped by protonation within acidic compartments of the cell, thereby inducing osmotic swelling of these compartments resulting in vacuole development. Internalization and transport to the late endosomal compartment of the anion-selective channels formed by VacA increase the anionic permeability of this compartment. In turn, this enhances the V-ATPase proton pumping activity leading, in the presence of weak bases like ammonia, to an increased accumulation of osmotically active ions such as NH_4_^+^. This finally leads to increased water influx and swelling [13,73].

Florey et al. [28] observed, in wild-type and Atg13-knockout MEFs, that 2 h exposure to VacA and ammonia caused Atg5-dependent LC3-II generation, with recruitment of GFP-LC3 to the typical single-membrane vacuoles induced by VacA. Such an effect was completely abolished by treatment with bafilomycin A_1_, a selective inhibitor of V-ATPase that blocks VacA-induced vacuole development [74]. In contrast, during autophagy, by disrupting the autophagic flux, bafilomycin A_1_ treatment leads to cellular accumulation of autophagosomes and, thus, of LC3-II. The finding by Florey et al. [28] is thus a further example of noncanonical functions of Atg proteins; namely, the participation of individual Atg factors in processes other than autophagy [29]. This should not be confused with noncanonical autophagy, that is, an autophagic process that may occur independently of one or more components of the Atg protein system [29]. Florey et al. [28] suggested that this type of LC3-II generation might account for most of the LC3 lipidation acutely induced by VacA. Although the exact function of LC3-II in nonautophagosomal membranes is still unclear, previous reports suggest that such a protein would favor membrane–membrane fusion (reviewed in [28,75]). This fusogenic action could thus be of help in promoting the typical increase in size of VacA-induced vacuoles with time, until most of the cytoplasm is occupied by a few large vacuoles.

The relationship between connexin 43 (Cx43) and autophagy in VacA-induced cell death makes an already complex story even more complicated. In 2014, Cover and coworkers [76] demonstrated that the unique highest sensitivity of AZ-521 cells to VacA-dependent cell death was accounted for by their expression of Cx43. However, Cx43 did not serve as a VacA receptor nor show any direct interaction with the toxin. Other cell lines widely used in studying VacA, such as HeLa and AGS cells, lack Cx43. When Cx43 was expressed in HeLa cells, they became more susceptible to VacA-induced cell death [76]. Cx43 is a multispan transmembrane protein that assembles to form gap junctions at the plasma membrane, but also localizes to intracellular sites such as mitochondria [76,77]. Cx43 is involved in several cell functions, including death and survival, through several mechanisms, some of which independent of its channel activity (reviewed in [76]). In comparison to the other transmembrane proteins, Cx43 has a short half-life and its rapid turnover is contributed by its autophagy-dependent degradation [59,77,78]. Nevertheless, the relationship between Cx43 and autophagy seems to be more complex than previously believed: plasma-membrane-localized Cx43 is reported to downregulate autophagy constitutively through direct interaction with several autophagy-related proteins involved in autophagosome biogenesis [77]. This inhibitory effect of Cx43 can be suppressed by its internalization and autophagy-dependent degradation, allowing for sustained activation of autophagy in response, for instance, to nutritional stress (starvation) [77].

Based on Cover and coworkers’ observations [76], Yahiro et al. [59] investigated the molecular pathways linking VacA to Cx43 that cause cell death in AZ-521 cells. VacA did not affect Cx43 expression but, following VacA-dependent LC3-II generation, Cx43 accumulated in the cytoplasm, resulting in apoptotic cell death. Cx43 accumulation was apparently due to inhibition of its degradation caused by dysfunctional autophagy. VacA increased Cx43 localization in endosomal/autophagic vesicles, but not in mitochondria where it may cause apoptosis. Confocal microscopy demonstrated colocalization of Cx43 with VacA and LC3 puncta; however, unlike LC3, both Cx43 and VacA were localized in cholesterol-rich, detergent-resistant membrane microdomains (lipid rafts). According to Yahiro et al. [59], Cx43 localization in lipid rafts might favor its resistance to degradation in autolysosomes. Nevertheless, Gauthier et al. [79] reported that VacA was typically associated with lipid rafts throughout its intracellular trafficking, from the plasma membrane to the late endosomal compartment. Apparently, this localization did not affect autophagic degradation of the toxin [49]. VacA-induced LC3-II generation was independent of Cx43, and, in VacA-treated cells, Atg16L1 silencing suppressed LC3-II generation, Cx43 increase, and PARP cleavage. In agreement with previous findings [58], VacA-induced LC3-II generation and Cx43 cytoplasmic accumulation were dependent on the channel-forming activity of the toxin because both were inhibited by chloride channel blockers. Surprisingly, silencing of LRP1 receptor was, on the contrary, ineffective in blocking LC3-II generation or Cx43 cytoplasmic accumulation induced by VacA. Silencing of other VacA receptors, such as such as RPTPα and β and fibronectin, did not affect VacA-induced LC3-II generation and Cx43 increase [59]. These data suggest that the action of VacA on autophagy and apoptosis relies also on a receptor and pathway different from the LRP1-dependent ones previously described. Sphingomyelin is one of the main constituents of lipid rafts and acts as a VacA receptor [80]. It is also an important determinant of the signaling pathway controlling the clathrin-independent endocytic mechanism through which VacA is taken up by epithelial cells [81]. Sphingolipids have recently emerged as critical regulators of apoptosis and autophagy [82].

According to Yahiro et al. [59], such an LRP1-independent pathway would involve GSH/ROS dysregulation, which is then followed by Rac1 activation and ERK phosphorylation. This then triggers Cx43 internalization and trafficking to autolysosomes where, however, Cx43 is not degraded. This impaired autophagic degradation of Cx43 results in increased cell death by apoptosis [59]. Yahiro et al. [59] also observed Cx43 accumulation in the gastric mucosa of *H. pylori*-infected patients in comparison to healthy individuals, thus suggesting a role of VacA-dependent alteration of Cx43 turnover in *H. pylori*-induced gastric pathogenesis.

## 5. Conclusions

The data I have tried to summarize above consistently show that, among *H. pylori* virulence factors, the VacA toxin plays a key role in the modulation of host cell autophagy exerted by the bacterium during infection of human gastric mucosa. Nevertheless, we are still far from a definitive description of the molecular mechanisms through which VacA affects autophagy, and of the consequences of such activity on the overall pathogenic action of *H. pylori*. Indeed, many important questions remain to be answered unequivocally (Table 1) and a few discrepancies in the data are emerging from a comparative analysis of the literature. Most of these discrepancies may derive from the use of different host cell models, different *H. pylori* strains and/or VacA preparations, and different incubation/treatment protocols.

As emphasized by Deen et al. [14], the design of definitive experiments to establish the dynamics and specific mode of action of VacA in affecting autophagy remains a key challenge. This requires an integrated technical approach, systematically combining light and especially electron microscopy with biochemistry and molecular biology. Indeed, TEM and ultrastructural immunocytochemistry remain a gold standard for careful time-dependent analysis of autophagy [26], even though they are more technically demanding in comparison to other approaches such as LC3-II generation analysis by Western blotting or fluorescence microscopy.

For instance, the relationship between autophagy and the action of VacA on the late endosomal/lysosomal compartment, which is at the intersection of the endocytic and autophagic pathways, deserves to be further investigated. In other words, VacA-induced vacuoles could finally find a well-defined role in the overall pathogenic action of *H. pylori* as key players in the autophagy modulation caused by the bacterium.

In all likelihood, the relationship between VacA and autophagy will thus continue to be a fascinating and rewarding subject for future studies.

## Figures and Tables

**Table 1:** Main questions still waiting for definitive answers.

What are the detailed mechanisms of the different VacA actions on the overall autophagic pathway?
Why is the channel-forming activity of VacA crucial for triggering autophagy? How does VacA decrease intracellular GSH level? Is this the result of a signal transduction pathway triggered at the plasma membrane by VacA interaction with its receptor(s) or is toxin internalization required? What is the trafficking pathway followed by VacA to enter autophagosomes? How is it connected to the well-known endocytic pathway followed by the toxin to reach the late endosomal compartment? Do defective autolysosomes with a reduced proteolytic activity, whose accumulation is caused by VacA, somehow differ from the well-known hybrid endolysosomal compartment, whose osmotic swelling leads to the typical VacA-dependent cell vacuolation? What are the exact timing and related molecular mechanisms through which an “acute” exposure to VacA stimulates autophagy, whereas a “chronic” one disrupt it? Does VacA-induced autophagy follow a canonical or noncanonical pathway? Does VacA also induce LC3-II recruitment to single-membrane phagosomes, thus triggering LC3-associated phagocytosis? May autophagy-independent LC3-II recruitment to single-membrane endolysosomal compartment (apparently triggered by the VacA-dependent osmotic imbalance of this compartment) have at least in part affected the reported results on the autophagic activity of VacA? Does VacA-induced mitochondrial damage also trigger mitophagy? If not, why? How is CagA (selectively?) targeted by the autophagic machinery for degradation? Does CagA accumulate only in autophagy-resistant CD44v9-positive cells or also in CD44v9-negative surface gastric epithelial cells in which prolonged VacA exposure disrupts the autophagic flux? Does VacA-induced impaired degradation of Cx43 in autolysosomes derive from Cx43 localization in lipid rafts or, rather, from the reduced proteolytic activity of autolysosomes caused by the toxin? What is the relationship between autophagy and apoptosis in VacA-treated cells? Is apoptosis the result of the stimulation or, on the contrary, of the disruption of autophagy induced by the toxin?
